# Inside the Mechanism of Action of Three Pyrazole Derivatives in Human Platelets and Endothelial Cells

**DOI:** 10.3390/antiox12020216

**Published:** 2023-01-17

**Authors:** Chiara Brullo, Eleonora Russo, Silvano Garibaldi, Paola Altieri, Pietro Ameri, Silvia Ravera, Maria Grazia Signorello

**Affiliations:** 1 Department of Pharmacy, Section of Medicinal Chemistry, University of Genova, 16132 Genova, Italy; 2 Department of Internal Medicine, University of Genova, 16132 Genova, Italy; 3 Cardiovascular Disease Unit, IRCCS Ospedale Policlinico San Martino, 16132 Genova, Italy; 4 Department of Experimental Medicine, University of Genova, 16132 Genova, Italy; 5 Department of Pharmacy, Biochemistry Lab, University of Genova, 16132 Genova, Italy

**Keywords:** human platelets, oxidative stress, oxidative phosphorylation, endothelial cells, antiproliferative activity, pyrazole, inflammation

## Abstract

In the effort to obtain multitarget compound interfering with inflammation, oxidative stress, and tumorigenesis, we synthesized a small library of pyrazole compounds, selecting **4a**, **4f**, and **4g** as the most noteworthy being IC_50_ against platelet ROS production induced by thrombin of about 10 µM. The in vitro antioxidant potential of the three molecules was evaluated, and since they show a remarkable antioxidative activity, their effect on several parameter indicative of oxidative status and on the efficiency of the aerobic metabolism was tested. The three molecules strongly inhibit superoxide anion production, lipid peroxidation, NADPH oxidase activity and almost restore the oxidative phosphorylation efficiency in thrombin-stimulated platelet, demonstrating a protective effect against oxidative stress. This effect was confirmed in endothelial cell in which **4a**, **4f**, and **4g** show an interesting inhibition activity on H_2_O_2_-stimulated EA.hy926 cells. At last, antiproliferative activity of **4a**, **4f**, and **4g** was submitted to a large screening at the NCI. The molecules show interesting anticancer activity, among them the most remarkable is **4g** able to strongly inhibit the proliferation of both solid tumor and leukemia cells lines. In conclusion, all the three newly synthetized pyrazoles show remarkable antioxidant and antiproliferative effect worthy of further study.

## 1. Introduction

Reactive oxygen species (ROS) play an important role in cell life being involved in physiological and pathological processes [1,2], regulating different signaling pathways, and controlling both cell proliferation and differentiation [3,4]. Oxidative stress, after ROS overproduction or dysfunction of the endogenous antioxidant system, can lead to damaged cells, the oxidation of proteins, lipids, and DNA, and cell function alterations [5]. Since ROS are reported to mediate several pathogenic conditions such as inflammation, neuroinflammation, tissue damage, and neurodegenerative diseases such as Alzheimer’s disease, cancer, atherosclerosis diabetes, as well as aging [6,7,8,9,10,11,12], ROS level control is of great importance to avoid undesired and damaging reactions [13].

It is known that platelets are strictly implicated in inflammatory processes, releasing inflammatory cytokines during activation/aggregation [14] and ROS at the site of the vascular injury [15], that could contribute to tumorigenesis [16]. Human platelets are activated by ROS released not only by polymorphonuclear leukocytes and red blood cells [17], but also by themselves upon stimulation by agonists [18,19,20,21,22]. So, human platelets could represent a fast, low-cost, and easy-to-use biological model to elucidate molecular mechanisms implicated in ROS production.

**Figure 1:**
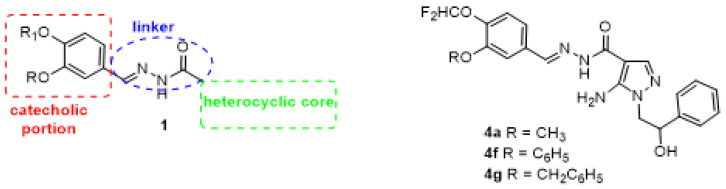
General structure of previous derivatives 1 and **4a**, **4f** and **4g**.

With the aim to obtain new molecules able to act on different targets involved in inflammation and cancer onset, we recently designed and synthesized a series of hybrid compounds 1 in which a heterocyclic core (pyrazole or imidazo-pyrazole) is linked to a catecholic portion through an acylhydrazone chain (Figure 1). Particularly, pyrazole molecules showed good antioxidant activity, being able to block ROS production in neutrophils and human platelets [23].

Among the newly synthetized library, the most active compounds in blocking ROS production in platelet resulted **4a**, **4f**, and **4g** (Figure 1), for which IC_50_ values, 10.1, 8.6, and 9.5 µM, respectively, were reported (Table 1 and ref. [23]). Interestingly, in these compounds, catechol portion was substituted by a difluoromethoxy group in para position 4 and by a smaller (-OCH_3_ in **4a**) or bigger and embedded (phenoxy and benzyloxy for **4f** and **4g** respectively) substituents in meta position.

As these three molecules have a different steric hindrance (particularly comparing **4a** with **4f** and **4g**), we evaluated in vitro antioxidant potential, then we have tested their effect on several parameter indicative of platelet oxidative status, i.e., superoxide anion production, lipid peroxidation, and NADPH oxidase activity. Their effect on aerobic metabolism efficiency, evaluated in terms of ATP synthesis and oxygen consumption rate, were also measured both in human platelets as well in endothelial cells EA,hy926 since **4a**, **4f**, and **4g** demonstrated ROS inhibition effect in these cells. At last, a preliminary screening of their antiproliferative activity on different cancer cell lines of the newly synthetized pyrazoles was performed.

## 2. Materials and Methods

### 2.1. Materials

2,2-diphenyl-1-picrylhidrazyl radical (DPPH), 6-hydroxy-2,5,7,8-tetramethylchroman-2-carboxylic acid (Trolox), ADP, apyrase, bovine serum albumin, butylated hydroxytoluene, cytochrome C, glutamine, dithiotreitol (DTT), DMEM, DMSO, FBS, L-lactic dehydrogenase (EC 1.1.1.27), leupeptin, malate, NAD^+^, NADH, PGE_1_, penicillin, phenylmethylsulfonyl fluoride (PMSF), protease inhibitor cocktail (Cat. N° P8340), pyruvate, streptomycin, succinate, superoxide dismutase (SOD), thiobarbituric acid (TBA), thrombin and all chemicals were from Sigma-Aldrich, St. Louis, MO, USA. CellROX^®^ Deep Red Reagent was from Life Technologies Corporation, Thermo Fisher Scientific, Waltham, MA, USA, and 96-well plates from Euroclone, Milan, Italy. ATP bioluminescence assay kit CLSII and ATP standard solution were from Roche, Switzerland.

### 2.2. In Vitro Antioxidant Activity (DPPH Assay)

The antioxidant activity was measured by the DPPH antioxidant assay. The assay is based on the bleaching rate of the stable radical DPPH [24]. Briefly, ca 3 mg of single compound was dissolved with methanol then 0.1 mL of this solution was mixed with 3.9 mL of DPPH methanol solution (65 µM). Absorbance was measured at 517 nm after reacting for 30 min in the dark. Linear calibration curve was obtained using Trolox standards (range between 20 to 200 mg/L, R^2^ = 0.9988). The result was calculated as Trolox equivalents in mg/L and the percentage of antioxidant activity (AA%) was calculated from the ratio of decreasing absorbance of sample solution (A_0_ − As) to absorbance of blank DPPH solution (A_0_), as expressed in Equation (1) [25].

AA% = [(A_0_ − As)/A_0_] × 100 (1)

### 2.3. Blood Collection and Preparative Procedures

Freshly drawn venous blood from healthy volunteers of the “Centro Trasfusionale, Ospedale San Martino” in Genoa was collected into 130 mM aqueous trisodium citrate anticoagulant solution (9:1). The donors claimed to have not taken drugs known to interfere with platelet function during two weeks prior to blood collection and gave their informed consent. Washed platelets were prepared centrifuging whole blood at 100× *g* for 25 min. To the obtained platelet-rich plasma (PRP) 4 mU/mL apyrase and 4 µM PGE_1_ were added. PRP was then centrifuged at 1100× *g* for 15 min. Pellet was washed once with pH 5.2 ACD solution (75 mM trisodium citrate, 42 mM citric acid and 136 mM glucose), centrifuged at 1100× *g* for 15 min and then resuspended in Ca^2+^-free HEPES buffer containing 145 mM NaCl, 5 mM KCl, 1 mM MgSO_4_, 10 mM glucose, 10 mM Hepes (pH 7.4). The reported IC50 value is the molar concentration of the compound able to obtain 50% inhibition of the maximal effect induced by the agonist and is calculated by the percentage of inhibition that is the inhibition of the maximal effect measured in the presence of the agent compared with that measured in a control sample containing saline, carried out under the same conditions.

### 2.4. Superoxide Anion Assay in Human Platelet

The production of superoxide anion was measured by mean of the difference between total and SOD-inhibitable cytochrome C reduction as described [18,26] with light modifications. Washed platelets (5.0 × 10^8^/mL), preincubated with saline or the compounds for 10 min at 37 °C in the presence of 100 µM cytochrome C and 300 U SOD, if present, were challenged with thrombin. Incubation was stopped by putting samples in ice. Samples were sedimented by centrifugation at 12,000× *g* for 8 min and reduced cytochrome C was measured in the supernatant by spectrophotometry at 550nm, in a Beckman DU530 (Brea, CA, USA) spectrophotometer, with molar extinction coefficient of 21,100 M^−1^ cm^−1^.

### 2.5. Lipid Peroxidation Measurement in Human Platelet

Lipid peroxidation was quantified by measuring thiobarbituric acid reactive substances (TBARS) as described [27] with light modifications. Washed platelets (5.0 × 10^8^/mL), preincubated with saline or the compounds for 10 min at 37 °C in the presence of butylated hydroxytoluene, was stimulated with thrombin. Incubation was stopped by cooling the samples in an ice bath in the presence of an equal volume of 20% trichloroacetic acid in 0.6 N HCl. One volume of supernatant obtained after 12,000× *g* for 5 min centrifugation was mixed with 0.2 volume of 0.12 M TBA in 0.26 M Tris (pH 7.0) and incubated for 30 min at 70 °C. The TBARS produced were assayed spectrophotometrically at 532 nm, in a Beckman DU530 spectrophotometer, with molar extinction coefficient of 156,000 M^−1^ cm^−1^.

### 2.6. NADPH Oxidase Activity Assay in Human Platelet

The enzymatic activity of NADPH oxidase was assessed spectrophotometrically in platelet homogenates by measuring the reduction of cytochrome C at 550 nm. Briefly, washed platelets (1.0 × 10^9^/mL), added to 10 µg/mL leupeptin, 1 mM PMSF, 100 µM DTT and 1/100 dilution protease inhibitor cocktail, were sonicated twice for 15 s and then centrifuged at 14,000× *g* for 10 min. Aliquots of the obtained supernatant, preincubated with saline or compounds for 10 min at 37 °C, were treated with thrombin. Incubation was stopped by cooling samples in ice and NADPH oxidase activity was assayed as reported [18]. Protein concentration was measured by Lowry method with bovine serum albumin as standard protein [28].

### 2.7. Tests to Assay Platelet Viability in Human Platelet

To check up platelet viability upon drug treatment the efficiency of the glycolytic pathway was measured by the production of L-lactate according to Hohorst [29]. To verify the membrane, damage the activity of lactic dehydrogenase released from platelets was quantified by the method of Vassault [30].

### 2.8. Antioxidant Activity on Endothelial Cells

EA.hy926 human endothelial cells (ATCC^®^ CRL-2922™) were cultured in DMEM supplemented with 10% FBS, 1% Glutamine, 1% penicillin/streptomycin [31]. Intracellular ROS production was evaluated on endothelial cells by CellROX^®^ Deep Red Reagent. Briefly, EA.hy926 endothelial cells were pretreated in 96-well plates with tested compounds for 60 min, stimulated for further 60 min with 50 μM H_2_O_2_ and then CellROX^®^ Reagent was added at a final concentration of 5 μM. CellROX^®^ loaded cells were incubated for 30 min at 37 °C, then the live cell nuclear reagent Hoechst 33,342 at 1 μg/mL was added and incubated for further 15 min. Afterwards cells were washed three times with PBS and read at ex/em 620/680 for CellROX^®^ Deep Red Reagent and at ex/em 360/485 for Hoechst 33,342 in a Spark multimode microplate Reader (Tecan Italia S.r.I., Milan, Italy). Results reported are CellROX^®^ to Hoechst fluorescence ratio.

### 2.9. Aerobic Metabolism in Human Platelets and EAhy926 Cell Line

Washed platelets (1.0 × 10^8^/mL) were preincubated with saline or compounds, stimulated at 37 °C with thrombin for 10 min and, at the end of incubation, samples were cooled in ice bath. Regarding EA.hy926, the human endothelial cells were incubated for a total of 90 min with **4a**, **4f**, or **4g**. After the first 30 min of incubation, 50 µM H_2_O_2_ was added to induce oxidative stress. For both platelet and EA.hy926 cells, the oxygen consumption rate (OCR) was measured in a closed chamber at 37 °C, by an amperometric O_2_ electrode (Unisense, Aarhus, Denmark). For each experiment, 10 µg of platelets total proteins or 10^5^ endothelial cells were resuspended in a medium containing 137 mM NaCl, 5 mM KH_2_PO_4_, 5 mM KCl, 0.5 mM EDTA, 3 mM MgCl_2_ and 25 mM Tris, pH 7.4 and permeabilized with 0.03% digitonin for 10 min. To stimulate the complexes I, III and IV or complexes II, III and IV, 10 mM pyruvate + 5 mM malate + 0.1 mM ADP or 20 mM succinate + 0.1 mM ADP were added, respectively [32,33].

To measure the aerobic ATP synthesis, the Fo-F1 ATP synthase activity was tested by luminometric analysis on digitonin-permeabilized platelets or endothelial cells, according to the procedure described for the OCR measurements. 10 µg of platelets total protein or 10^5^ endothelial cells were added to the incubation medium, containing 10 mM Tris (pH 7.4), 50 mM KCl, 1 mM EGTA, 2 mM EDTA, 5 mM KH_2_PO_4_, 2 mM MgCl_2_, 0.6 mM ouabain, 0.040 mg/mL ampicillin, 0.2 mM di-adenosine-5′penta-phosphate and the respiratory substrates 10 mM pyruvate + 5 mM malate or 20 mM succinate. To stimulate the ATP synthesis 0.1 mM ADP was added. The ATP synthesis was measured using the luciferin/luciferase ATP bioluminescence assay kit CLSII, on a Promega GloMax^®^ 20/20 Luminometer. ATP standard solutions were used in the range 10^−10^–10^−7^ M for calibration [32,33].

To evaluate the oxidative phosphorylation (OxPhos) efficiency in energy production, P/O value has been calculated. P/O value represents the ratio between the number of ATP molecules synthesized with aerobic respiration and the number of oxygen atoms consumed in the process. Efficient mitochondria, when stimulated with pyruvate and malate, have a P/O value around 2.5, whereas when stimulated with succinate the P/O value must be around 1.5 [34,35]. A P/O ratio lower than 2.5 for pyruvate and malate or lower than 1.5 for succinate means that some of the oxygen is not used for energy production but contributes to ROS formation.

### 2.10. Anti-Proliferative Activity

Testing was performed by the Developmental Therapeutics Program, Division of Cancer Treatment and Diagnosis, National Cancer Institute (Available online: http://dtp.cancer.gov (accessed on 30 November 2022)).

### 2.11. Statistical Analysis

Data are mean ± SD of at least two independent experiments, each performed at least in duplicate. Statistical comparisons between two groups were made through the multiple unpaired t-test. To compare multiple groups one-way ANOVA followed by Dunnett’s post hoc test was used. Statistical significance was defined as *p* < 0.05.

## 3. Results

### 3.1. In Vitro Antioxidant Activity (DPPH)

The antioxidant activity (AA%), reported in Table 2, was calculated by the Trolox linear calibration curve, in which the equation is y = −0.2018x + 93.374, and calculated by (1). The R^2^ value (0.9988) indicated the high sensitivity of the analytical test and highlighted an excellent correlation between the two variables considered. All the tested compounds showed a good antioxidant activity, among them **4g** was the molecule with the most marked AA%, while **4a** and **4f** were found to have a value of about half compared to the previous ones.

### 3.2. Human Platelets Oxidative Status

**Figure 2:**
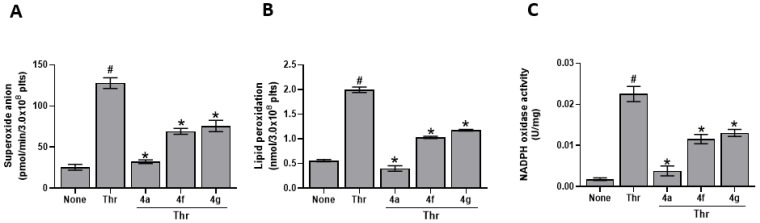
Effect of **4a**, **4f**, **4g** on superoxide anion production, lipid peroxidation and NADPH oxidase activity. Washed platelets (1.0 × 10^8^/mL), preincubated 10 min with saline or 20 µM **4a**, **4f**, or **4g** were stimulated for 10 min with 0,1U/mL thrombin (Thr). Superoxide anion formation (panel (**A**)), lipid peroxidation (panel (**B**)) and NADPH oxidase activity (panel (**C**)) were determined as detailed in Methods. Data are the mean ± SD of four experiments carried out in duplicate. Multiple unpaired *t* test: # *p* < 0.0001 vs. None; * *p* < 0.0001 vs. Thr.

Since it was reported [23] that **4a**, **4f**, and **4g** can inhibit ROS production and aggregation in human platelets stimulated by thrombin, we tested their effect on other parameters indicative of platelets oxidative status. As reported in Figure 2, **4a**, **4f**, and **4g** strongly inhibit superoxide anion formation (Figure 2A) and lipid peroxidation (Figure 2B) in human platelets stimulated by thrombin. **4a** seems to be the more effective since IC_50_ values (Table 3) are slightly lower than **4f** and **4g** in all cases. Since NADPH oxidase is one of the major sources of ROS in the cells, we evaluated the effect of **4a**, **4f**, and **4g** on NADPH oxidase activity induced by thrombin (Figure 2C and Table 3). The molecules inhibit the activity of the enzyme and the results obtained are in strict correlation with those obtained on superoxide anion production and lipid peroxidation, being R^2^ = 0.9963 and R^2^ = 0.9995, respectively.

### 3.3. Tests to Assay Platelet Viability

Treating human platelets with different concentrations of **4a**, **4f**, and **4g** (range 1–1000 μM), we observed no significative difference as compared with control in lactate production or in lactic dehydrogenase release during 10 min of incubation at 37 °C (data not shown).

### 3.4. Antioxidant Activity on Endothelial Cells

**Figure 3:**
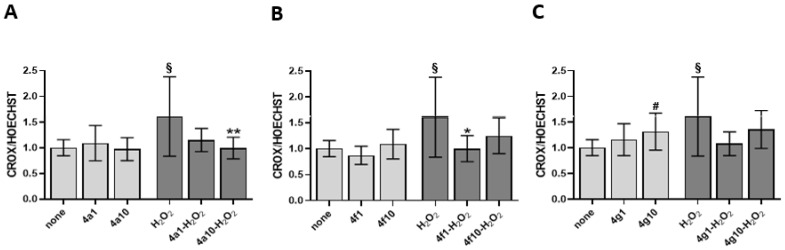
ROS production assay on endothelial cells. ROS assay evaluated as CellROX to Hoechst fluorescence ratio on endothelial cells pretreated with **4a** panel (**A**), **4f** panel (**B**) and **4g** panel (**C**) at 1 and 10 µM and challenged with 50 µM H_2_O_2_. 4a1, 4f1, 4g1 are the compounds tested at 1 µM while 4a10, 4f10, 4g10 at 10 µM. Data are the mean ± SD of two experiments carried out in triplicate. One-way ANOVA-Dunnett’s post hoc test: # *p* < 0.05 vs. none, ** *p* < 0.01, * *p* < 0.05 vs. H_2_O_2_. Multiple unpaired *t* test: § *p* < 0.005 vs. none.

The antioxidant activity of the compounds **4a**, **4f**, and **4g** was evaluated in Eahy926 EA.hy926 endothelial cell line, as a somatic cell model. H_2_O_2_ has been used as a pro-oxidizing agent, inducing superoxide anion production in endothelial cells through NADPH oxidase activation [36]. As shown in Figure 3, regarding ROS production inhibition in endothelial cells, pretreatment with compound **4a** was associated with the strongest antioxidant activity both at concentration of 1 and 10 µM. Compounds **4f** and **4g** showed a different activity on endothelial cells depending on the concentration, with a significant antioxidant activity at 1 µM concentration. While at the lowest concentration these two compounds exerted a significant antioxidant protective activity, at the highest concentration the effect was moderately pro-oxidant, especially for **4g**.

### 3.5. Aerobic Metabolism in Platelets

**Figure 4:**
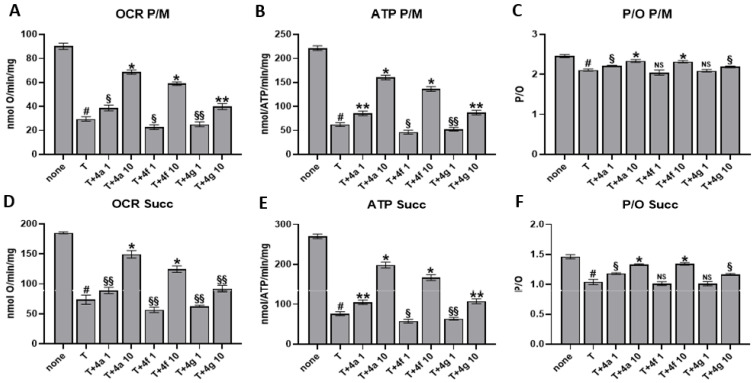
Platelet aerobic metabolism. Washed platelets (1.0 × 10^8^/mL) preincubated with saline or 1 µM or 10 µM newly drugs, were stimulated for 10 min at 37 °C with 0.1 U/mL thrombin (T). Panels (**A**,**B**) show the oxygen consumption rate (OCR) and the aerobic ATP synthesis, respectively, measured in the presence of pyruvate + malate while panels (**D**,**E**) in the presence of succinate. Panels (**C**–**F**) reports P/O values as OxPhos efficiency markers. Data are the mean ± SD of at least four experiments. Multiple unpaired *t* test: # *p* < 0.01 vs. none; * *p* < 0.0001, ** *p* < 0.0005, § *p* < 0.005, §§ *p* < 0.05 vs. T, NS: not significant.

Besides NADPH oxidase activation, OxPhos is considered the principal source of ROS. Thus, the effects of the three compounds were evaluated on OCR, aerobic ATP synthesis, and OxPhos efficiency in thrombin-treated platelets. As reported in Figure 4, thrombin led to a dramatic decrease in OCR and ATP synthesis, also causing an evident uncoupling between energy synthesis and respiration, both in the presence of pyruvate + malate or in the presence of succinate. By contrast, the drug-pretreatment reversed OxPhos inhibition in a dose-dependent manner. In detail, the most remarkable seems to be **4a**, which produces, in the pyruvate + malate experimental condition, activation of about 250% versus thrombin. Further, **4f** and **4g** appear less active reaching about 200% and 140% of activation, respectively. When the complex II pathway is stimulated, the drug reversal effect is still noteworthy although less evident than in pyruvate + malate conditions, being 4a activation of about 200% and 4f, 4g of about 170% and 130%, respectively.

### 3.6. Aerobic Metabolism in EA.hy926 Cell Line

**Figure 5:**
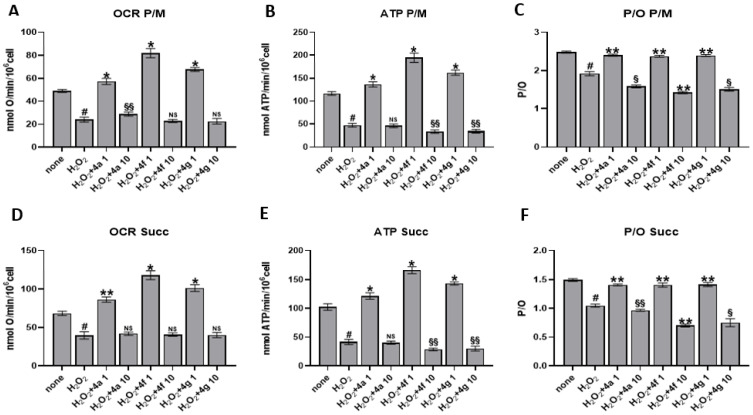
EA.hy926 human endothelial cells aerobic metabolism. Mitochondrial metabolism has been evaluated in EA.hy926 after the treatment with 1 µM or 10 µM **4a**, **4f**, or **4g** in the presence of 50 µM H_2_O_2_ as oxidative stimulus. Panels (**A**,**B**) show the oxygen consumption rate (OCR) and the aerobic ATP synthesis, respectively, measured in the presence of pyruvate + malate (P/M) while panel (**D**,**E**) reports the same analyses in the presence of succinate (Succ). Panels (**C**,**F**) reports P/O values obtained in the presence of both respiratory substrates as OxPhos efficiency markers. Data are the mean ± SD of at least four experiments. Multiple unpaired *t* test: # *p* < 0.01 vs. none; * *p* < 0.0001, ** *p* < 0.0005, § *p* < 0.005, §§ *p* < 0.05 vs. H_2_O_2_, NS: not significant.

The effect of the three new compounds was also tested on the aerobic metabolism of endothelial cells after treatment with 50 µM H_2_O_2_ per 60 min, a pro-oxidative stimulus. Data reported in Figure 5 show that hydrogen peroxide addition causes a decrease in oxygen consumption and an even more marked decrease in ATP synthesis, causing an uncoupling between respiration and energy production. However, these effects are reversed by pretreatment with the three compounds at the lowest dose (1 µM), increasing mitochondrial function above that of the untreated sample not subjected to pro-oxidative stimulus. In detail, after pyruvate + malate or succinate addition, compound **4f** showed a higher effect on OxPhos compared to **4a** and **4g**. Furthermore, it should be noted that, despite the increase in mitochondrial activity, the system is perfectly coupled, as shown by the P/O values. In contrast, the 10 µM treatment of the new compounds causes a further decrease in oxygen consumption and ATP synthesis, increasing the uncoupling between the function of respiratory complexes and ATP synthase.

### 3.7. Antiproliferative Activity Evaluation

Compounds **4a**, **4f**, and **4g** were submitted to a large screening to evaluate their anticancer activity (National Cancer Institute, Germantown MD, USA). This is a very broad analysis of the anti-proliferative action, considering the most common cancers in adults, including both highly metastatic and less aggressive cell lines. In detail, compounds were screened on 60 tumor cell lines (most common cancer cell lines in adults, including highly metastatic and aggressive ones) at high dose (10^−5^ M) (SRB Cytotoxicity Assay) (Available online: http://dtp.cancer.gov) (accessed on 30 November 2022). Compound **4a** showed a weakly antiproliferative activity (25–30% of growth percent, Table 4), particularly against non-small cell lung cancer, central nervous system cancer cells and renal cancer; on the contrary, pyrazoles **4f** and **4g** evidenced some antiproliferative activity, particularly against different solid tumors as non-small-cell lung cancer, colon, ovarian, renal, prostate, melanoma and CNS tumor cell lines, (Table 4). Benzyloxy substituted 4g was able to block proliferation also of different leukaemia cell lines.

## 4. Discussion

Our aim was to define the mechanism of **4a**, **4f**, and **4g**, three newly synthesized pyrazole derivatives selected among a large number of compounds for their antioxidant and antiaggregating activity (Figure 1 and Table 1) [23]. Firstly, we verified antioxidant properties of **4a**, **4f**, and **4g** by DPPH scavenging assay that is one of the most economical methods to measure in vitro antioxidant activity. This assay is based on the conversion of DPPH to DPPHH, which results in attenuation of the absorbance value at 517 nm, showing a good in vitro antioxidant activity by the three drugs. Since, as previously reported [23], the compounds demonstrated a noteworthy IC_50_ against platelet ROS production induced by thrombin, we tested their ability to inhibit other parameters indicative of oxidative status in human platelets, such as superoxide anion production, lipid peroxidation and NADPH oxidase activity. It is known that ROS include oxygen ions, free radicals, and peroxides. Most intracellular ROS are derived from superoxide anion, which is considered critical for initiating changes in cellular signaling events along with hydrogen peroxide [37,38]. Moreover, ROS can react with lipids, proteins, and DNA causing irreversible damage in their structure and function [39,40,41]. Thus, the consequent lipid peroxidation, strictly connected to cellular oxidative stress, induces various pathogenic intracellular signals leading to cellular dysfunctions. The three compounds, **4a**, **4f**, and **4g** induce strong and significative inhibition of both superoxide anion formation and lipid peroxidation in thrombin-stimulated platelets, and, thus, they could exert an interesting protective effect. Among them, **4a** seems to be lightly more effective since the IC_50_s reported are lightly lower than **4f** and **4g** (Table 3). One of the main sources of ROS is NADPH oxidase, ubiquitous in all cells in which several isoforms have been described. In detail, human platelets express NADPH oxidase1 and NADPH oxidase2 [42] that play different roles in platelet activity [43]. Data reported in Figure 2C show that **4a**, **4f**, and **4g** inhibit NADPH oxidase activity induced by thrombin in strict correlation to the diminished superoxide anion formation (R^2^ = 0.9963) and lipid peroxidation (R^2^ = 0.9995). Thus, in thrombin stimulated platelets pretreated with **4a**, **4f**, and **4g**, the reduced activity of NADPH oxidase seems to be one of the modulators of the antioxidant effect of the three molecules. Since oxidative stress could be also dependent on alteration of aerobic metabolism, we have tested the effect of the three pyrazoles on oxygen consumption and on the ATP synthesis in thrombin-stimulated platelets. As previously reported [44], thrombin impairs mitochondrial complex I, diminishing electron flow through the electron transport chain and the consequent ATP production, thus reducing the OxPhos efficiency and increasing ROS production. The three molecules can almost restore control values in both OCR and ATP synthesis. In detail, among the three compounds, **4a** seems to be the most effective, while **4f** and **4g** appear less active. In addition, the pretreatment with **4a**, **4f**, and **4g** ameliorates the OxPhos coupling, explaining their antioxidant effect despite the increment of mitochondria activity. In fact, under decoupling conditions, the OxPhos is less efficient, but the respiratory complexes produce more free radicals because they are unlocked from ATP synthase activity. In other words, pretreatment with **4a**, **4f**, and **4g** improves both the functionality and efficiency of aerobic metabolism of thrombin-treated platelets, reducing the production of oxidative stress.

Relevant are the biological data obtained in endothelial cells, particularly for compound **4a**, resulted the most active at 1 µM as well as at 10 µM concentration. The behavior of **4f** and above all **4g** is slightly different, with the latter in particular showing better antioxidant activity at low concentrations (1 µM) and a slightly pro-oxidant action at higher doses (10 µM) (Figure 5). It is known that H_2_O_2_, a pro-oxidizing agent, induces superoxide anion production through NADPH oxidase and the OxPhos uncoupling in endothelial cells [24,31,45]. However, as observed for platelets, treatment with 1 µM of **4a**, **4f**, and **4g** restores aerobic metabolism function and efficiency, even increasing it compared to the control treated with neither compounds nor H_2_O_2_. Therefore, it is possible to speculate that the antioxidant effect of **4a**, **4f**, and **4g** on endothelial cells subjected to oxidative stress depends not only on the restoration of OxPhos uncoupling but also on an increased energy availability to respond to oxidative damage via endogenous antioxidant defenses. On the other hand, Kowald and Kirkwood predicted that cells could use up to 55% of the total energy to repair and/or prevention of free radical and oxidative damage [46]. By contrast, the pre-treatment with 10 µM causes a further OCR and ATP synthesis reduction, and the increment of the uncoupling between energy production and respiration suggesting a hormetic effect. Hormesis consists of a biphasic response to a molecule based on concentration: low doses cause a beneficial effect, while high doses lead to toxic or inhibitory effects [47]. Beyond the **4a**, **4f**, and **4g** effects, only metformin displays a hormetic effect on mitochondrial function [48,49]. However, several dietary phytochemicals have demonstrated hormetic effects on several pathways involved in cellular redox regulation [50]. Further on the effect of higher compound doses, excessive antioxidant activity may be deleterious since free radicals at low concentration are necessary for cellular signaling and functions, stimulating also endogenous antioxidant defense. Pro-thrombotic and pro-inflammatory pathways recognize oxidative stress as a unifying mechanism leading to development of endothelial dysfunction, and cardiovascular disease, cancer with metastatic complications as well as metabolic and several other diseases. Thus, antioxidant pharmacological approach, besides being still debated for its clinical efficacy, appears to be a needed target for therapy when fine-tuned (with regard to both concentration and molecule choice) on specific pathologic condition.

The weak pro-oxidant activity displayed by compound **4g** in our model at the highest concentration used (10 µM) may be related to the highest anti-proliferative activity evidenced on tumor cells. In fact, particularly for compounds **4g**, antiproliferative activity against different solid tumors, as well as in leukemia cell lines, it is certainly noteworthy (Table 4). This issue should be further investigated to ascertain the useful concentration and conditions for the usage of these compounds. In addition, the hugely different steric hindrance of catechol moiety between **4a, 4f,** and **4g** could be responsible of different biological effect in platelets respect to endothelial cells. Catechol derivatives (in particular if decorated with smaller substituent as **4a**) are reported as phosphodiesterase 4 inhibitors (PDE4Is) [51]. While in platelets this isoform is absent, in endothelial cells this specific isoform seems to be present [52] and could be blocked more potently by **4a** respect to a more embedded catechol derivatives **4f** and **4g**.

## 5. Conclusions

In conclusion, these three newly synthesized compounds exert a protective effect against oxidative stress as they ameliorate the oxidative status in human platelets as well as in endothelial cells. Further, **4a** seems to be the most interesting since it shows the highest activity in both tested models. However, **4g** could be noteworthy too, since even if it shows a lesser antioxidant activity, it evidences a good antiproliferative action; this biological profile suggests for **4g** a multitarget behavior at intracellular level. The satisfactory results obtained, both as antiproliferative agents and antioxidant agents, make these compounds worthy of further study.

## Figures and Tables

**Table 1:** Inhibiting effect of com-pounds 4a, 4f and 4g on aggregation and reactive oxygen species production [23].

Cmpd.	Aggregation Inhibition IC_50_ (µM)	ROS Production Inhibition IC_50_ (µM)
**4a**	±0.5	±2.1
**4f**	±1.7	±2.2
**4g**	±1.2	±2.6

**Table 2:** Evaluation of antioxidant activity percent (AA%).* DPPH% = (As/A_0_) × 100: As is the sample absorbance and A_0_ is blank DPPH solution absorbance.

Cmpd.	DPPH% *	AA%
**4a**	90.3 ± 0.4	9.8 ± 0.4
**4f**	90.3 ± 0.3	9.7 ± 0.3
**4g**	81.9 ± 0.4	18.1 ± 0.4

**Table 3:** IC_50_ values.

Cmpd.	Superoxide Anion IC_50_ (µM)	Lipid Peroxidation IC_50_ (µM)	NADPH Oxidase Activity IC_50_ (µM)
**4a**	13.4 ± 0.4	12.3 ± 0.6	12.6 ± 0.5
**4f**	21.8 ± 0.7	20.5 ± 0.5	20.7 ± 0.4
**4g**	24.6 ± 0.6	23.8 ± 0.6	24.4 ± 0.6

**Table 4:** Cell growth percent values of pyrazoles **4a**, **4f**, and **4g** on different cancer cell lines at of 10^−5^ M concentration. For each compound, only cell lines with a growth percent values < 25% are indicated. Variation among triplicate was less than 10%.

Cmpd.	Cancer Cell Lines		Cell Growth Percent (%)
**4a**	Non small cell lung cancer	HOP-92	74.0
		NCI-H460	75.0
	CNS cancer	SNB-75	74.0
	Renal cancer	UO-31	68.0
	Leukaemia	RPMI-8226	49.0
	Colon Cancer	HCT-15	47.0
**4f**	CNS cancer	SNR-75	50.5
	Melanoma	SKMEL-5	37.6
	Renal cancer	UO-31	45.6
	Breast cancer	HS578T	47.3
	Leukaemia	CCRF-CEM	18.5
		MOLT-4	17.5
		K-562	27.3
		RPMI-8226	13.4
		SR	40.3
	Non-Small cell lung cancer	A549/ATCC	45.8
	Melanoma	LOXIMVI	21.1
		SK-MEL5	22.5
		UACC-62	23.9
	Ovarian cancer	IGROV-1	46.7
**4g**		OVCAR-3	34.9
		OVCAR-4	28.8
		OVCAR-8	16.6
		NCI/ADR-RES	37.3
	Renal cancer	ACHN	48.9
		CAKI-1	35.0
		SN12C	44.3
		UO-31	38.3
	Prostate cancer	PC-3	20.4
		DU-145	50.2
	Breast Cancer	MCF-7	30.3
		HST578T	22.2
		BT-549	33.8

## Data Availability

The data presented in this study are available in the article.
